# Three visual working memory representations simultaneously control attention

**DOI:** 10.1038/s41598-020-67455-y

**Published:** 2020-06-29

**Authors:** Michael J. King, Brooke N. Macnamara

**Affiliations:** Department of Psychological Sciences, Case Western Reserve University, 11220 Bellflower Road, Cleveland, OH 44106 USA

**Keywords:** Psychology, Human behaviour

## Abstract

How many items can we store in visual working memory while simultaneously conducting a visual search? Previous research has proposed that during concurrent visual search, only one visual working memory representation can be activated to directly influence attention. This previous research suggests that other visual working memory representations are “accessory items”, which have little direct influence on attention. However, recent findings provided evidence that not one, but two visual working memory representations can capture attention and interfere with concurrent visual search. We successfully replicate these findings, and further test whether the capacity of visual working memory during visual search extends to not two, but three representations that influence attention directly. We find evidence that three visual working memory representations can simultaneously control attention.

## Introduction

Whether looking for a parking spot in a garage, looking for car keys on a messy desk, or looking for a friend among a large crowd of people, people struggle with ignoring distractors during visual search. Many of the major theories of attention propose that during visual search, visual working memory deploys attention and biases perceptual processing toward memory items^1–3^. Visual working memory refers to our capacity to store and manipulate visual objects, features, and object–feature conjunctions in memory^4^.

Research suggests that the transfer of an item to visual working memory occurs automatically when attention is focused on an item^5^. Thus, the limited capacity of visual working memory during visual search is a direct result of the limited number of items that can be simultaneously attended. Researchers have suggested that humans, on average, can hold 3–5 items in working memory at a time^6,7^. However, other researchers claim that this amount is reduced when attentional resources are simultaneously being used (e.g., during visual search), suggesting that only one visual working memory representation can be active at a time in the focus of attention^8,9^.

Along these lines, other research suggests that there are two types of visual working memory representations^10^. One type is an *active memory item.* An active memory item has direct access to perception, and thus will influence attention during visual search. The other type refers to *accessory memory items*. Accessory memory items are passively stored in visual working memory, and exert little influence on attention during visual search.

According to some research^11^, only one item in visual working memory at a time can serve as the active memory item. Consistent with this, if memory items vary trial to trial, it is assumed that the *active memory item* consumes the only slot in visual working memory during visual search. Thus, an irrelevant stimulus that matches other accessory working memory contents does not interfere with concurrent visual search because only the active item biases attention^12^.

However, when the active memory item is constant from trial to trial, this representation is eventually transferred to long-term memory instead of being actively maintained in visual working memory. This allows another item in visual working memory to guide attention^13–15^. For example, if a person must remember to grab their keys on a repeated basis while searching for their car in a busy parking lot, the image of their car keys should eventually get transferred from active working memory, to a long-term memory representation, allowing an accessory memory item to fill the active memory slot.

Additionally, there is a fair amount of research on multiple target searches that report switch costs and limits to attentional selection with more than one search target, supporting the idea that only a single item in visual working memory can be active at a time^11,16–18^. For example, in two studies^16,17^, researchers asked participants to search for two targets among distractors simultaneously. They found only small costs associated with holding both items in visual working memory, but substantial costs when conducting a visual search for both objects. These results suggest that while more than one item can be stored in visual working memory in preparation for search, there seems to be a cost for actually searching for these representations, suggesting that participants can only activate one visual working memory item for search at a time.

If the hypothesis that a single active visual working memory representation biases attention during concurrent visual search is correct, this might reflect a fundamental bottleneck in human information processing^1,13^. However, recent findings demonstrating that irrelevant stimuli matching either of two visual working memory representation can involuntarily capture attention, suggest otherwise^19–21^. These findings show that visual search can be under simultaneous control of two visual working memory representations, in the form of single featured color stimuli^22^. However, while these color targets change from trial to trial, there are only two possible target colors. The limited possible target colors could cause the two visual working memory representations to quickly be transferred to participants’ long-term memories^13–15^. Therefore, these findings may not actually demonstrate two active representations in visual working memory.

Chen and Du^1^ investigated whether two visual working memory representations can simultaneously guide attention and interfere with concurrent visual search. They used unique memoranda with multiple features (color and texture) that varied from trial to trial. Thus, the items should theoretically be maintained as visual working memory representations and not transferred to long-term memory. Further, the authors used memoranda that were not the target of visual search. This method assumes that memoranda held in visual working memory are active if they capture attention during visual search for different targets, because accessory items should not guide attention. However, this method differs from past studies where the memoranda were also the search targets^16,17^.

Chen and Du^1^ found that two visual working memory representations captured attention and interfered with concurrent visual search^1^. In addition, each of these two visual working memory representations interfered with concurrent visual search as much as a single cued representation^1^. They concluded, that not one, but two visual working memory representations can be active memory items^1^.

### Present study

Findings from Chen and Du^1^ suggest that two visual working memory representations can capture attention and interfere with concurrent visual search. In our Experiment 1, we conduct a direct replication of Chen and Du’s^1^ Experiment 2. If a direct replication produces similar findings as the original study, this increases confidence in the results^23^. In our Experiment 2, we test whether two items represent the full capacity of active visual working memory representations during visual search, or whether three visual working memory representations can capture attention and interfere with visual search.

## Experiment 1

### Introduction

**Figure 1:**
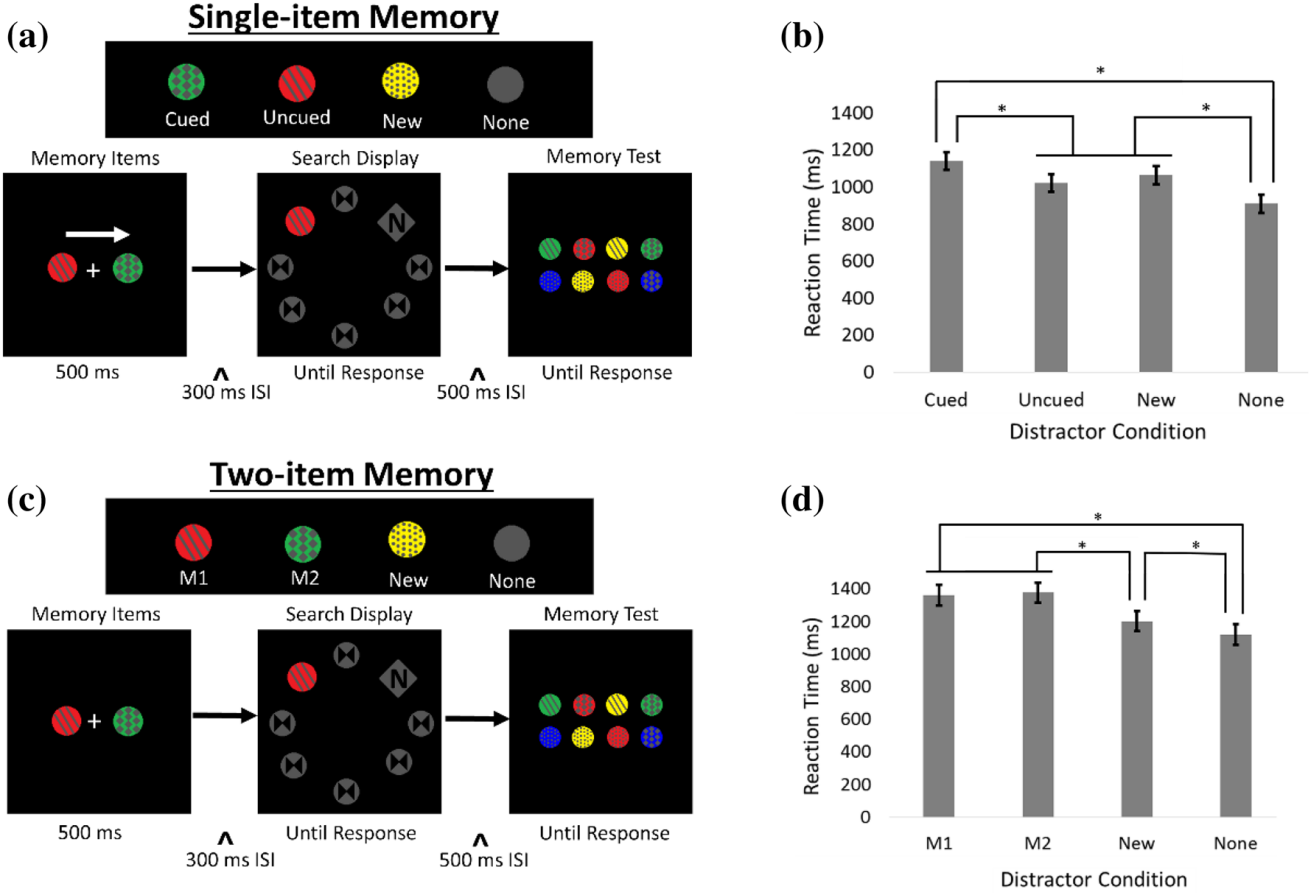
Schematic of trial events and results for Experiment 1. (**a**) Trial display with four different possible distractor conditions per trial for the single-item memory condition (the “Cued distractor”: the circle’s color and texture were identical to the Cued memorized item; the “Uncued distractor” [shown here]: the circle’s color and texture were identical to the uncued memorized item; the “New distractor”: the distractor circle was a different color and texture from the circles presented at the beginning of the trial; the “None distractor”: no distractor circle appeared, all seven circles were gray). (**b**) RTs for the search display as a function of distractor condition in the single cued memory item condition. (**c**) Trial display with four different possible distractor conditions per trial for the two-item memory condition (the “M1 distractor” [shown here]: the circle’s color and texture were identical to the memorized item on the left; the “M2 distractor”: the circle’s color and texture were identical to the memorized item on the right; the “New distractor” and “None distractor” conditions were the same as their counterparts in the single-item memory condition). (**d**) RTs for the search display as a function of distractor condition in the two-item memory condition. Error bars indicate ± 1 standard errors of the mean.

In an exact replication of Chen and Du’s^1^ Experiment 2, we first tested whether we could reproduce the evidence that two items simultaneously held in visual working memory can bias attention during visual search. This study was pre-registered on the Open Science Framework (https://osf.io/8m4nj). See Fig. 1 for trial sequences.

### Results

See Tables 1 and 2 for descriptive statistics. In line with Chen and Du’s^1^ results, in the single-item memory condition, there was a main effect of distractor condition, *F*(3, 129) = 82.54, *p* < 0.001, *η*_*p*_^2^ = 0.66. Post-hoc follow-up pairwise comparisons with Bonferroni adjustments revealed that the RTs in the Cued distractor condition were significantly longer than the RTs of the Uncued distractor condition, *t*(43) = 8.6, *p* < 0.001, *d* = 1.29, the New distractor condition, *t*(43) = 6.4, *p* < 0.001, *d* = 0.98, and the None distractor condition, *t*(43) = 14.5, *p* < 0.001, *d* = 1.86. The RTs in the Uncued distractor condition and the New distractor condition did not differ from each other, *t*(43) = − 2.7, *p* = 0.062. The RTs from both the Uncued distractor condition and the New distractor condition were significantly longer than the RTs from the None distractor condition, *t*(43) = 6.9, *p* < 0.001, *d* = 1.05, and, *t*(43) = 9.8, *p* < 0.001, *d* = 1.45, respectively. See Fig. 1b.

**Table 1:** Reaction times (s) for Experiment 1’s visual search test across all conditions.

Memory condition	Distractor condition	*Mean (SD)*
Single-item memory	Cued	1.14 (0.226)
	Uncued	1.02 (0.229)
	New	1.06 (0.215)
	None	0.91 (0.184)
Two-item memory	M1	1.36 (0.356)
	M2	1.37 (0.349)
	New	1.20 (0.297)
	None	1.11 (0.290)

**Table 2:** MCI value (s) for Experiment 1’s visual search test across all conditions.

Memory condition	Distractor condition	*Mean (SD)*
Single-item memory	MCI cued	0.31 (0.290)
Two-item memory	MCI M1	0.76 (0.770)
	MCI M2	0.70 (0.529)

Also in line with Chen and Du’s^1^ results, in the two-item memory condition, there was a main effect of distractor condition, *F*(3, 129) = 62.54, *p* < 0.001, *η*_*p*_^2^ = 0.59. Post-hoc follow-up pairwise comparisons with Bonferroni adjustments revealed that the RTs from the M1 distractor and the M2 distractor did not differ from one another (*p* = 0.137). The RTs from the M1 distractor and the M2 distractor were both significantly longer than the RTs of the New distractor condition, *t*(43) = 6.5, *p* < 0.001, *d* = 0.99, and *t*(43) = 7.0, *p* < 0.001, *d* = 1.06, respectively, and significantly longer than the RTs of the None distractor condition, *t*(43) = 9.0, *p* < 0.001, *d* = 1.34, and *t*(43) = 9.9, *p* < 0.001, *d* = 1.48, respectively. Additionally, the New distractor condition produced significantly longer RTs than the None distractor condition, *t*(43) = 4.9, *p* < 0.001, *d* = 0.76. See Fig. 1d.

A memory-capture index (MCI^1^) was calculated to measure the interference caused by distractors. The MCI is calculated by taking the difference between the mean reaction time of the cued condition and the new condition, divided by 0.5. This value is then multiplied by the sum of the mean reaction time from the cued condition and the new condition [MCI = (RTcue-RTnew)/0.5 × (RTcue + RTnew)].

If participants are merely alternating whether M1 or M2 is the sole active representation, then the combined MCI for M1 and M2 (M1 + M2) should not be significantly different than the MCI of a single cued memory item. Additionally, if only one visual working memory representation can be activated and there is a bias toward one of the memoranda, the MCI of one of them (M1 or M2) should be smaller than that for a single cued memory item.

However, if M1 and M2 in the two memory items condition are both being held in visual working memory, the combined MCI for M1 and M2 (M1 + M2) should be significantly larger than the MCI of a single cued memory item. The results of Experiment 1’s MCI analysis supported that two representations were being maintained: the combined MCI effect of the M1 and M2 distractors in the two-item memory condition was significantly larger than that of the cued distractor in the single-item memory condition (mean difference = 1.16, *p* < 0.001).

Due to confusing typesetting of the MCI equation in Chen and Du^1^, the equation could actually be interpreted in two different ways. One, is the way it is interpreted in the present study. It could also be that Chen and Du^1^ calculated the MCI by taking the difference in RTs (RTcue–RTnew) and dividing it by the mean of those RTs [(RTcue + RTnew)/2]. This would resemble an effect size calculation: the amount of capture normalized by the average RT in the task. Due to this confusion, we decided to calculate the MCI this second way in addition to the original interpretation. The analysis of this also supported that two representations were being maintained: the combined MCI effect of the M1 and M2 distractors in the two-item memory condition was significantly larger than that of the cued distractor in the single-item memory condition (mean difference = 0.162, p < 0.001).

## Experiment 2

### Introduction

**Figure 2:**
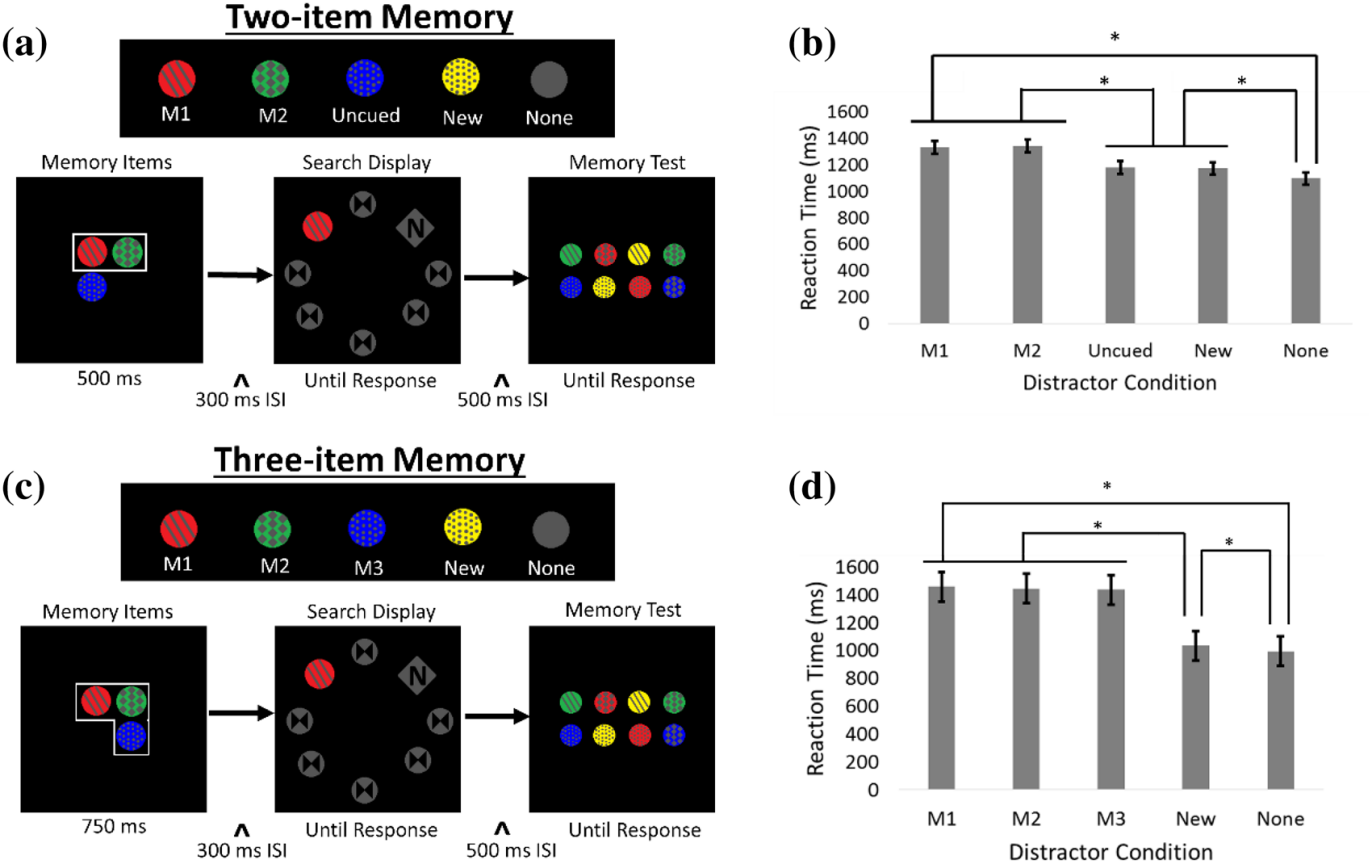
Schematic of trial events and results for Experiment 2. (**a**) Trial display with four different possible distractor conditions per trial for the two-item memory condition (the “M1 distractor” [shown here]: the circle’s color and texture were identical to the memorized item on the top left; the “M2 distractor”: the circle’s color and texture were identical to the memorized item on the top right; the “Uncued distractor”: the circle’s color and texture were identical to the uncued memorized item; the “New distractor”: the distractor circle was different from the circles presented at the beginning of the trial; the “None distractor”: no distractor circle appeared, all seven circles were gray). (**b**) RTs for the search display as a function of distractor condition in the two-item memory condition. (**c**) Trial display with five different possible distractor conditions per trial for the three-item memory condition (the “M1 distractor” [shown here], “M2 distractor”, “New distractor”, and “None distractor” are the same as in the two memory items condition; the “M3 distractor”: the circle’s color and texture were identical to the memorized item on the bottom). (**d**) RTs for the search display as a function of distractor condition in the three-item memory condition. Error bars indicate ± 1 standard errors of the mean.

In Experiment 1, we confirmed Chen and Du’s^1^ finding that two visual working memory representations can simultaneously control attention. However, it has not been tested whether two items represent the full active capacity of visual working memory during visual search. To follow up on these results, Experiment 2 investigated whether two items is the limit, or if the capacity can extend to three items in visual working memory that simultaneously control attention during visual search. See Fig. 2 for trial sequences (Table 3).

**Table 3:** Accuracy results for visual search and memory test in Experiment 1.

Condition	Mean visual search accuracy (SD)	Mean memory test accuracy (SD)
Single-item memory	0.97 (0.03)	0.76 (0.09)
Two-item memory	0.96 (0.03)	0.74 (0.08)

### Results

See Tables 4 and 5 for descriptive results. In the two-item memory condition, there was a main effect of distractor condition, *F*(4, 220) = 19.70, *p* < 0.001, *η*_*p*_^2^ = 0.26. Post-hoc follow up pairwise comparisons with Bonferroni adjustments revealed that the RTs of the M1 distractor and the M2 distractor did not differ significantly from one another, *t*(55) = -0.6, *p* = 0.235, and the RTs of both the M1 and M2 distractor conditions were significantly longer than the RTs of the Uncued distractor condition, *t*(55) = 2.9, *p* < 0.05, *d* = 0.39, and *t*(55) = 3.3, *p* < 0.01, *d* = 0.44 respectively, the New distractor condition, *t*(55) = 6.4, *p* < 0.001, *d* = 1.05, and *t*(55) = 7.9, *p* < 0.001, *d* = 1.14 respectively, and the None distractor condition, *t*(55) = 9.3, *p* < 0.001, *d* = 1.34, and *t*(55) = 10.4, *p* < 0.001, *d* = 1.40 respectively. Also, the Uncued and the New distractor conditions both produced significantly longer RTs than the None distractor condition, *t*(55) = 1.9, *p* < 0.05, *d* = 0.26, and *t*(55) = 4.8, *p* < 0.001, *d* = 0.77 respectively. See Fig. 2. This was the same pattern of results revealed in Experiment 1’s two-item memory condition (Table 6).

**Table 4:** Reaction times (s) for Experiment 2’s visual search test across all conditions.

Memory condition	Distractor condition	*Mean (SD)*
Two-item memory	M1	1.33 (0.354)
	M2	1.34 (0.343)
	Uncued	1.18 (0.332)
	New	1.17 (0.299)
	None	1.09 (0.184)
Three-item memory	M1	1.45 (0.249)
	M2	1.44 (0.234)
	M3	1.43 (0.205)
	New	1.04 (0.204)
	None	0.89 (0.222)

**Table 5:** MCI value (s) for Experiment 2′s visual search test across all conditions.

Memory condition	Distractor condition	*Mean (SD)*
Two-item memory	MCI M1	0.82 (1.07)
	MCI M2	0.87 (0.983)
Three-item memory	MCI M1	2.02 (1.11)
	MCI M2	2.06 (0.732)
	MCI M3	1.97 (1.33)

**Table 6:** Accuracy results for visual search and memory test in Experiment 2.

Condition	Mean visual search accuracy (SD)	Mean memory test accuracy (SD)
Two-item memory	0.92 (0.10)	0.81 (0.12)
Three-item memory	0.90 (0.12)	0.76 (0.09)

In the three-item memory condition, there was a main effect of distractor condition, *F*(4, 220) = 228.1, *p* < 0.001, *η*_*p*_^2^ = 0.80. Post-hoc follow-up pairwise comparisons with Bonferroni adjustments revealed that the RTs of the M1, M2, and M3 distractors were not significantly different from one another, all *p*s > 0.500. The RTs of the M1, M2, and M3 distractor conditions were significantly longer than the RTs of the New distractor condition, *t*(55) = 13.8, *p* < 0.001, *d* = 1.15; *t*(55) = 28.1, *p* < 0.001, *d* = 1.24; *t*(55) = 17.5, *p* < 0.001, *d* = 1.19, respectively, and the None distractor condition, *t*(55) = 20.7, *p* < 0.001, *d* = 1.27; *t*(55) = 32.7, *p* < 0.001, *d* = 1.36; *t*(55) = 25.1, *p* < 0.001, *d* = 1.29 respectively. The New distractor condition produced significantly longer RTs than the None distractor condition, *t*(55) = 10.4, *p* < 0.001, *d* = 0.59. See Fig. 2.

A memory-capture index (MCI^1^) was again calculated to measure the interference caused by distractors. The results of Experiment 2’s MCI analysis suggests that all three representations were being maintained: the combined MCI effect of the M1, M2, and M3 distractors in the three-item memory condition was significantly larger than the combined MCI effect of the M1 and M2 distractors in the two-item memory condition (mean difference = 4.37, *p* < 0.001).

Due to confusing typesetting of the MCI equation in Chen and Du^1^, the equation could actually be interpreted in two different ways. One, is the way it is interpreted in the present study. It could also be that Chen and Du^1^ calculated the MCI by taking the difference in RTs (RTcue–RTnew) and dividing it by the mean of those RTs [(RTcue + RTnew)/2]. This would resemble an effect size calculation: the amount of capture normalized by the average RT in the task. Due to this confusion, we again decided to calculate the MCI this second way in addition to the original interpretation, just as in Experiment 1. The results from this analysis also supported the finding that all three representations were being maintained: the combined MCI effect of the M1, M2, and M3 distractors in the three-item memory condition was significantly larger than the combined MCI effect of the M1 and M2 distractors in the two-item memory condition (mean difference = 0.747, p < 0.001).

## Discussion

In two experiments we demonstrated that multiple items held in visual working memory can capture attention and interfere with visual search. In the first experiment, we replicated Chen and Du’s^1^ experiment demonstrating that not one, but two items held in visual working memory can capture attention. In the second experiment, we demonstrate that not two, but three items held in visual working memory can capture attention. These findings are in contrast to proposals that only one visual working memory representation can influence attention directly at a time during visual search^10–12^.

The stimuli were designed such that the memoranda were selected from twelve possible stimuli and the stimuli varied trial to trial. This stimuli design means it is unlikely participants are committing the memoranda to long-term memory. Further, the results do not support the argument that the memoranda are simply being alternatively activated to influence visual search, such that multiple items are sharing the same slot available for an active memory item. Instead, the MCI results suggest that at least three representations can be simultaneously activated in visual working memory.

Future research is needed to test whether three items is the limit, or if four, five, or more items represent the capacity of active items in visual working memory during visual search. Another direction for future research is to test whether individuals vary in their active visual working memory capacities during visual search, just as individuals vary in their overall working memory capacity^24,25^. Using the current paradigm, individuals with greater active visual working memory capacity would show greater deficits in their visual search (longer RTs), but higher memory accuracy than individuals who presumably could not hold as many items in active memory, and would therefore have less interference.

Further, future research is needed to determine whether the multiple maintained memoranda guiding attention are active in the same way other researchers^16^ have defined active items: as target templates. If so, the items should be able to serve as simultaneous search targets. Given previous research, this outcome seems unlikely. Our differing results from prior research that uses multiple target templates, suggests that our capacity to actively maintain irrelevant items *during* visual search is higher than our capacity to actively maintain relevant items *for* a visual search. This is perhaps surprising given research demonstrating interference from dual tasks, due either to a hypothesized specific resource limitation^26^ or general resource limitation^27,28^. Our results are also at odds with research that asserts that visual attention is a single resource^29^.

Rather, our findings (and Chen and Du’s findings) support the idea that dual tasks and visual search engage two distinct attentional resources^30^. That is, our findings suggest that multiple items being maintained in visual working memory, but unrelated to the single target of a visual search, can drive attention during that search. By contrast, it appears that multiple targets of a visual search cannot simultaneously guide attention during that search. This suggests that the visual working memory capacity for active items in dual tasks is higher than the visual working memory capacity for active items for search targets. In other words, simultaneous active maintenance of memoranda unrelated to search appears to be three, whereas active maintenance of target templates during search may be just one.

An alternative argument is that the items guiding attention are not truly active, but demonstrate increased functionality of accessory items. That is, active items imply search targets. Therefore, our results do not suggest distinct resources, but that maintained items are not as “passive” as previously thought. In this case, future research is needed to update hypotheses where a defining trait of accessory items is their lack of ability to capture attention during visual search e.g.,^12^.

A final argument is that items need not be target templates, only to actively guide attention during visual search, to be considered active items. Assuming this definition, visual working memory does not appear to reflect a bottleneck in information processing as previously thought. Rather than a single active visual working memory representation biasing attention during concurrent visual search, we provided evidence that not one, or two, but three visual working memory representations can simultaneously capture attention during concurrent visual search. Thus, the number of unrelated items that can interfere with our search while we look for a parking spot in a garage, car keys on a messy desk, or a friend among a large crowd of people, is higher than previously thought.

## Methods

### Experiment 1

#### Participants

Chen and Du’s^1^ Experiment 2 included 40 participants. We recruited 46 participants through the Case Western Reserve University SONA system subject pool based on our preregistered data collection stopping rule. Our stopping rule, based on standards set by previous research^1^, was to stop data collection at the end of the week following the 40th participant. Following our exclusion criteria, two participants’ data were removed because their accuracy on the memory task was below 60%. The study was performed in accordance with the Declaration of Helsinki and was approved by the Case Western Reserve University IRB review board. Participants gave their written informed consent to participate in the study. They received course credit for their participation.

#### Materials and equipment

The experiment was controlled using the open source application PsychoPy^31^ on a 2014 9,020 all-in-one Dell Optiplex desktop computer with a 15-in. CRT monitor (90-Hz refresh rate) at a viewing distance of approximately 60 cm.

#### Visual task description and procedure.

##### Memoranda

Each trial began with two circles (each with a radius of 0.6°) appearing on the screen for 500 ms that the participants were asked to remember. Each circle consisted of one of 12 possible color-texture combinations. The four colors included: red (RGB: 250, 20, 0), green (RGB: 0, 170, 0), yellow (RGB: 220, 200, 20), or blue (RGB: 0, 90, 200). Each circle also consisted of one of three types of texture (checkerboard, striped, or reticulation).

In the one-memory-item condition, a gray arrow (RGB: 85, 85, 85; 0.8° in width, 1.6° in length) pointing either to the right or left, indicated to participants that they were supposed to only memorize that specific circle. In the two-memory-item condition, no arrow appeared, indicating that participants were to memorize both circles. Each presented circle was randomly selected from a pool of 12 possible combinations of four colors and three textures.

##### Search

Following the appearance of the two circles, the screen was blank for 300 ms, and then participants were presented with a search display. The search display consisted of a gray diamond (1.2° in size) and seven circle distractors (each with a radius of 0.6°). They were placed on the rim of an imaginary circle (with a radius of 8°), which was centered on the fixation. The diamond contained a black target letter which could be either an “N” or an “M” (0.38° in size). Participants were instructed to indicate whether the diamond contained an “N” or “M” as fast as possible, by hitting the “N” or “M” key on the keyboard. The search display remained on the screen until participants selected “N” or “M.” Each gray circle on the search display contained a symbol resembling an hourglass. One of the seven circles served as a distractor (see Fig. 1). There were four distractor types. See Table 7.

**Table 7:** Experiment 1 distractor types.

Distractor condition	Description
**Single-item memory condition**	
Cued distractor	Circle’s color and texture are the same as the cued item
Uncued distractor	Circle’s color and texture are the same as the uncued item
New distractor	Circle’s color and texture are different from the cued and uncued items
No distractor	No circle with color or texture is present
**Two-item memory condition**	
M1 distractor	Circle’s color and texture are the same as the item presented on the left
M2 distractor	Circle’s color and texture are the same as the item presented on the right
New distractor	Circle’s color and texture are different from the cued and uncued items
No distractor	No circle with color or texture is present

##### Memory test

Following the search display, a blank screen appeared for 500 ms. Next, a probe screen appeared where the participants were tasked with responding whether or not one of a set of 8 probe circles matched one of the memoranda. Probe circles could share the same color but differ in texture or share the same texture but differ in color as the memorized item, therefore participants could not use a single feature for memory task. In the single-item memory condition, the cued item was only presented on a half of the trials, while the uncued item never appeared as a probe circle. However, in the two-memory item condition, in which participants were instructed to memorize both items, the M1 and M2 items were present in the probes with equal probability for 50% of the trials. They never occurred in the probe display simultaneously.

##### Task procedure

The order of the two memory conditions was counter-balanced across participants. Each participant completed two single-item memory sets and two two-item memory sets. Conditions were counterbalanced. There were 24 practice trials per condition, and 192 experimental trials per condition (48 trials per set). Each distractor condition (Cued, Uncued, New, None; M1, M2, New, None) was presented an equal number of times within each memory set, in a random order.

### Experiment 2

#### Participants

Based on our data collection stopping rule, sixty participants were recruited through the Case Western Reserve University SONA system subject pool. Our stopping rule, based on the increased complexity of this experiment relative to Experiment 1, was to stop data collection at the end of the week following the 60th participant. However, two participants were excluded because their accuracy on the memory task was below 60%, and two participants were excluded due to uncooperative behavior during the experiment. The study was approved by the Case Western Reserve University IRB review board. Participants gave their written informed consent to participate in the study. They received course credit for their participation.

#### Materials and equipment

The experiment was controlled using the open source application PsychoPy^31^ on a 2014 9,020 all-in-one Dell Optiplex desktop computer with a 15-in. CRT monitor (90-Hz refresh rate) at a viewing distance of approximately 60 cm.

#### Visual task description and procedure

The methods are identical to the methods of Experiment 1 except as described below.

Memoranda. In the two-memory-item condition, three memoranda were presented at the beginning of each trial and were displayed for 500 ms. An outline surrounded two of the three circles indicating that participants were supposed to only memorize those specific circles (see Fig. 2). The outline always surrounded the upper two circles, and participants were instructed to only memorize the outlined circles. In the three-memory-item condition, three memoranda were presented at the beginning of each trial and were displayed for 750 ms. An outline surrounded all of the circles indicating that participants were supposed to memorize all the circles.

Search. The search was identical to Experiment 1 except for the distractor conditions. See Table 8. In the M1 distractor condition, the circle’s color and texture was the same as the memorized item appearing in the top left position. In the M2 distractor condition, the circle’s color and texture was the same as the memorized item in the top right position. In the M3 condition, the circle’s color and texture was the same as the memorized item in the lower position. In the new-distractor condition, the circle was a combination of color and texture that was not part of the memoranda. In the no-distractor condition, all seven circles were gray.

**Table 8:** Experiment 2 distractor types.

Distractor condition	Description
**Two-item memory condition**	
M1 distractor	Circle’s color and texture are the same as the item presented on the top-left
M2 distractor	Circle’s color and texture are the same as the item presented on the top-right
Uncued distractor	Circle’s color and texture are the same as the uncued item
New distractor	Circle’s color and texture are different from the M1, M2, and uncued items
No distractor	No circle with color or texture is present
**Three-item memory condition**	
M1 distractor	Circle’s color and texture are the same as the item presented on the top-left
M2 distractor	Circle’s color and texture are the same as the item presented on the top-right
M3 distractor	Circle’s color and texture are the same as the item presented on the bottom
New distractor	Circle’s color and texture are different from the cued and uncued items
No distractor	No circle with color or texture is present
